# 1,1′-Di-*n*-butyl-4,4′-bipyridinium 2.375-bromido-1.625-chloridocadmate

**DOI:** 10.1107/S1600536811000080

**Published:** 2011-01-12

**Authors:** Wei-Juan Wang, Jun-Ming Yue, Yun-Yin Niu, Seik Weng Ng

**Affiliations:** a Department of Chemistry, Zhengzhou University, Zhengzhou 450052, People’s Republic of China; b Department of Chemistry, University of Malaya, 50603 Kuala Lumpur, Malaysia

## Abstract

The title salt, (C_18_H_26_N_2_)[CdBr_2.375_Cl_1.625_], consists of non-inter­acting cations and tetra­hedral cadmate(II) anions. The halogen atoms are all disordered, the bromine components being in 0.9035 (17):0.0965 (17), 0.6581 (18):0.3419 (18), 0.5019 (19):0.4981 (19) and 0.6847 (19):0.3153 (18) ratios. The aromatic rings of the cation are twisted by 25.0 (1)°.

## Related literature

For the synthesis of the cation, see: Hou *et al.* (2005 ▶). For a similar disordered tetrahalogenidocadmate, see: Liu *et al.* (2007 ▶).
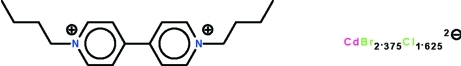


## Experimental

### 

#### Crystal data

(C_18_H_26_N_2_)[CdBr_2.375_Cl_1.625_]
*M* *_r_* = 630.20
Monoclinic, 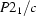

*a* = 8.6969 (5) Å
*b* = 16.6024 (10) Å
*c* = 16.6881 (10) Å
β = 104.936 (1)°
*V* = 2328.2 (2) Å^3^
*Z* = 4
Mo *K*α radiation
μ = 5.21 mm^−1^
*T* = 100 K
0.30 × 0.30 × 0.10 mm

#### Data collection

Bruker SMART APEX diffractometer
Absorption correction: multi-scan (*SADABS*; Sheldrick, 1996 ▶) *T*_min_ = 0.304, *T*_max_ = 0.624
21634 measured reflections
5354 independent reflections
4487 reflections with *I* > 2σ(*I*)
*R*_int_ = 0.045

#### Refinement

*R*[*F* ^2^ > 2σ(*F* ^2^)] = 0.031
*wR*(*F* ^2^) = 0.078
*S* = 1.02
5354 reflections
234 parameters
5 restraints
H-atom parameters constrained
Δρ_max_ = 1.49 e Å^−3^
Δρ_min_ = −1.27 e Å^−3^

### 

Data collection: *APEX2* (Bruker, 2009 ▶); cell refinement: *SAINT* (Bruker, 2009 ▶); data reduction: *SAINT*; program(s) used to solve structure: *SHELXS97* (Sheldrick, 2008 ▶); program(s) used to refine structure: *SHELXL97* (Sheldrick, 2008 ▶); molecular graphics: *X-SEED* (Barbour, 2001 ▶); software used to prepare material for publication: *publCIF* (Westrip, 2010 ▶).

## Supplementary Material

Crystal structure: contains datablocks global, I. DOI: 10.1107/S1600536811000080/xu5137sup1.cif

Structure factors: contains datablocks I. DOI: 10.1107/S1600536811000080/xu5137Isup2.hkl

Additional supplementary materials:  crystallographic information; 3D view; checkCIF report

## Figures and Tables

**Fig. 1.**
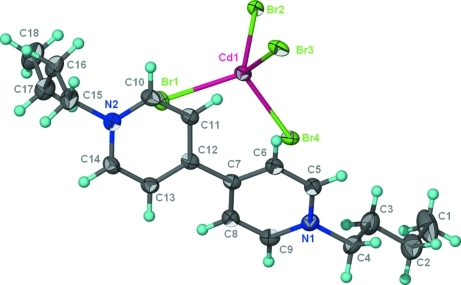
Thermal ellipsoid plot (Barbour, 2001) of [C18H26N2]2+ [Br2.375Cl1.625Cd]2- at the 70% probability level; hydrogen atoms are drawn as spheres of arbitrary radius. The halogen atoms are all disordered, and are represented as bromide atoms.

## supplementary crystallographic information

### Comment

The class of 1,1'-dialkyl-4,4'-bipyridinium bromides represents a class of ammonium salts that are excellent directing regents for the construction of metal–organic architectures. In a previous study, the reaction of a similar salt, 1,1'-(propane-1,3-diyl)dipyridinium dibromide, with cadmium dichloride, yielded the salt as a dibromidodichlorodocadmate; the halogen atoms are all disordered. In the present study, the reaction of 1,1'-dibutyl-4,4'-bipyridinium dibromide with cadmium dichloride yielded a similarly disordered cadmate counterion whose bromine:chloride ratio is 2.375:1.625 (Scheme I, Fig. 1).

### Experimental

1,1'-Dibutyl-4,4'-bipyridinium dibromide was synthesized by using a literature method (Hou *et al.*, 2005). 1-Bromobutane (30 mmol, 4.11 g) and 4,4&prime;-bipyridyl (10 mmol, 1.56 g) were dissolved in acetonitrile (20 ml). The solution was heated at 343–353 K for 48 h. The yellow precipitate that formed was collected and recrystallized from a methanol/ether mixture to give a white powder (4.2 g, 90% yield.)

A methanol solution (10 ml) of the dibromide salt (0.43 g, 1.0 mmol) was added to a solution of cadmium dichloride (0.184 g, 1.0 mmol) dissolved in an DMF/H_2_O (4:1) mixture (10 ml) to precipitiate a white solid. This dissolved when DMF was added. The solution was filtered and then set aside for the growth of colorless crystals (40% yield) after a week. The crystals are not soluble in common solvents.

### Refinement

Hydrogen atoms were placed in calculated positions (C—H 0.95 to 0.99 Å) and were included in the refinement in the riding model approximation, with *U*(H) set to 1.2–1.5*U*_eq_(C).

Each halogen site is occupied by a mixture of chlorine and bromine atoms. For each site, the temperate factors for the major and minor occupants were restrained to be identical. As the total occupancy of the bromine atoms refined to nearly 2 3/8, the sum occupany was then fixed as exactly 2 3/8. The final difference Fourier had a peak at 1.83 Å from H2b and a hole at 0.88 ° from Cd1

### Crystal data

**Table:**

(C_18_H_26_N_2_)[CdBr_2.375_Cl_1.625_]	*F*(000) = 1227
*M**_r_* = 630.20	*D*_x_ = 1.798 Mg m^−^^3^
Monoclinic, *P*2_1_/*c*	Mo *K*α radiation, λ = 0.71073 Å
Hall symbol: -P 2ybc	Cell parameters from 8220 reflections
*a* = 8.6969 (5) Å	θ = 2.4–28.3°
*b* = 16.6024 (10) Å	µ = 5.21 mm^−^^1^
*c* = 16.6881 (10) Å	*T* = 100 K
β = 104.936 (1)°	Prism, colorless
*V* = 2328.2 (2) Å^3^	0.30 × 0.30 × 0.10 mm
*Z* = 4	

### Data collection

**Table:**

Bruker SMART APEX diffractometer	5354 independent reflections
Radiation source: fine-focus sealed tube	4487 reflections with *I* > 2σ(*I*)
graphite	*R*_int_ = 0.045
ω scans	θ_max_ = 27.5°, θ_min_ = 1.8°
Absorption correction: multi-scan (*SADABS*; Sheldrick, 1996)	*h* = −11→11
*T*_min_ = 0.304, *T*_max_ = 0.624	*k* = −21→21
21634 measured reflections	*l* = −21→21

### Refinement

**Table:**

Refinement on *F*^2^	Primary atom site location: structure-invariant direct methods
Least-squares matrix: full	Secondary atom site location: difference Fourier map
*R*[*F*^2^ > 2σ(*F*^2^)] = 0.031	Hydrogen site location: inferred from neighbouring sites
*wR*(*F*^2^) = 0.078	H-atom parameters constrained
*S* = 1.02	*w* = 1/[σ^2^(*F*_o_^2^) + (0.037*P*)^2^ + 2.9972*P*] where *P* = (*F*_o_^2^ + 2*F*_c_^2^)/3
5354 reflections	(Δ/σ)_max_ = 0.001
234 parameters	Δρ_max_ = 1.49 e Å^−^^3^
5 restraints	Δρ_min_ = −1.27 e Å^−^^3^

### Fractional atomic coordinates and isotropic or equivalent isotropic displacement parameters (Å^2^)

**Table:**

	*x*	*y*	*z*	*U*_iso_*/*U*_eq_	Occ. (<1)
Cd1	0.40196 (3)	0.424706 (15)	0.232277 (14)	0.01974 (7)	
Br1	0.22214 (4)	0.35248 (2)	0.10618 (2)	0.02326 (11)	0.9035 (17)
Br2	0.23823 (5)	0.48953 (3)	0.32488 (2)	0.02183 (13)	0.6581 (18)
Br3	0.62807 (6)	0.34583 (3)	0.32995 (3)	0.02465 (15)	0.4981 (19)
Br4	0.53176 (7)	0.53958 (4)	0.17615 (3)	0.02306 (17)	0.3153 (19)
Cl1	0.22214 (4)	0.35248 (2)	0.10618 (2)	0.02326 (11)	0.0965 (17)
Cl2	0.23823 (5)	0.48953 (3)	0.32488 (2)	0.02183 (13)	0.3419 (18)
Cl3	0.62807 (6)	0.34583 (3)	0.32995 (3)	0.02465 (15)	0.5019 (19)
Cl4	0.53176 (7)	0.53958 (4)	0.17615 (3)	0.02306 (17)	0.6847 (19)
N1	0.8621 (3)	0.47579 (17)	0.09803 (18)	0.0213 (6)	
N2	0.4249 (3)	0.11942 (17)	0.06874 (17)	0.0197 (6)	
C1	0.9057 (6)	0.7653 (3)	0.1760 (3)	0.0485 (12)	
H1A	0.9725	0.8134	0.1798	0.073*	
H1B	0.8931	0.7519	0.2311	0.073*	
H1C	0.8011	0.7758	0.1383	0.073*	
C2	0.9844 (5)	0.6951 (2)	0.1432 (3)	0.0393 (10)	
H2A	0.9944	0.7081	0.0869	0.047*	
H2B	1.0926	0.6866	0.1795	0.047*	
C3	0.8885 (5)	0.6192 (2)	0.1401 (3)	0.0326 (9)	
H3A	0.7786	0.6288	0.1062	0.039*	
H3B	0.8832	0.6049	0.1969	0.039*	
C4	0.9603 (4)	0.5501 (2)	0.1036 (2)	0.0264 (8)	
H4A	0.9689	0.5651	0.0475	0.032*	
H4B	1.0689	0.5393	0.1385	0.032*	
C5	0.8900 (4)	0.4246 (2)	0.1624 (2)	0.0227 (7)	
H5	0.9711	0.4363	0.2113	0.027*	
C6	0.8021 (4)	0.3558 (2)	0.1579 (2)	0.0222 (7)	
H6	0.8222	0.3202	0.2040	0.027*	
C7	0.6840 (4)	0.3373 (2)	0.08722 (19)	0.0186 (7)	
C8	0.6557 (4)	0.3926 (2)	0.0221 (2)	0.0237 (7)	
H8	0.5749	0.3823	−0.0272	0.028*	
C9	0.7442 (4)	0.4616 (2)	0.0293 (2)	0.0246 (7)	
H9	0.7222	0.4999	−0.0145	0.029*	
C10	0.4928 (4)	0.1502 (2)	0.1435 (2)	0.0222 (7)	
H10	0.4818	0.1232	0.1919	0.027*	
C11	0.5781 (4)	0.2205 (2)	0.1510 (2)	0.0199 (7)	
H11	0.6269	0.2416	0.2044	0.024*	
C12	0.5935 (4)	0.26102 (19)	0.0800 (2)	0.0180 (6)	
C13	0.5245 (4)	0.2266 (2)	0.0037 (2)	0.0209 (7)	
H13	0.5354	0.2519	−0.0456	0.025*	
C14	0.4404 (4)	0.1562 (2)	−0.0009 (2)	0.0214 (7)	
H14	0.3927	0.1330	−0.0535	0.026*	
C15	0.3217 (4)	0.0467 (2)	0.0626 (2)	0.0266 (8)	
H15A	0.3777	0.0048	0.1015	0.032*	
H15B	0.2982	0.0246	0.0057	0.032*	
C16	0.1663 (5)	0.0697 (2)	0.0838 (3)	0.0317 (8)	
H16A	0.1011	0.0205	0.0824	0.038*	
H16B	0.1915	0.0912	0.1410	0.038*	
C17	0.0703 (4)	0.1309 (2)	0.0261 (2)	0.0313 (8)	
H17A	0.0397	0.1083	−0.0307	0.038*	
H17B	0.1372	0.1790	0.0252	0.038*	
C18	−0.0791 (5)	0.1565 (3)	0.0506 (3)	0.0444 (11)	
H18A	−0.1375	0.1961	0.0107	0.067*	
H18B	−0.0495	0.1805	0.1062	0.067*	
H18C	−0.1467	0.1093	0.0509	0.067*	

### Atomic displacement parameters (Å^2^)

**Table:**

	*U*^11^	*U*^22^	*U*^33^	*U*^12^	*U*^13^	*U*^23^
Cd1	0.02436 (13)	0.01957 (13)	0.01644 (12)	−0.00087 (9)	0.00734 (10)	−0.00126 (9)
Br1	0.0256 (2)	0.0214 (2)	0.02208 (19)	0.00007 (14)	0.00474 (15)	−0.00461 (14)
Br2	0.0222 (2)	0.0263 (2)	0.0179 (2)	−0.00361 (16)	0.00688 (16)	−0.00281 (16)
Br3	0.0347 (3)	0.0242 (3)	0.0167 (2)	0.0036 (2)	0.0096 (2)	−0.00047 (19)
Br4	0.0288 (3)	0.0255 (3)	0.0168 (3)	−0.0041 (2)	0.0094 (2)	0.0004 (2)
Cl1	0.0256 (2)	0.0214 (2)	0.02208 (19)	0.00007 (14)	0.00474 (15)	−0.00461 (14)
Cl2	0.0222 (2)	0.0263 (2)	0.0179 (2)	−0.00361 (16)	0.00688 (16)	−0.00281 (16)
Cl3	0.0347 (3)	0.0242 (3)	0.0167 (2)	0.0036 (2)	0.0096 (2)	−0.00047 (19)
Cl4	0.0288 (3)	0.0255 (3)	0.0168 (3)	−0.0041 (2)	0.0094 (2)	0.0004 (2)
N1	0.0219 (14)	0.0195 (15)	0.0245 (15)	−0.0002 (11)	0.0099 (12)	−0.0017 (12)
N2	0.0199 (14)	0.0162 (14)	0.0226 (14)	0.0030 (11)	0.0050 (11)	0.0009 (11)
C1	0.057 (3)	0.028 (2)	0.046 (3)	0.004 (2)	−0.012 (2)	−0.006 (2)
C2	0.044 (2)	0.030 (2)	0.040 (2)	−0.0057 (19)	0.004 (2)	0.0020 (18)
C3	0.0293 (19)	0.029 (2)	0.042 (2)	−0.0049 (16)	0.0124 (17)	−0.0086 (18)
C4	0.0270 (18)	0.0233 (19)	0.0318 (19)	−0.0068 (14)	0.0126 (16)	−0.0020 (15)
C5	0.0216 (16)	0.0253 (19)	0.0212 (16)	−0.0018 (14)	0.0059 (14)	−0.0021 (14)
C6	0.0228 (17)	0.0250 (19)	0.0195 (16)	−0.0005 (14)	0.0071 (14)	−0.0011 (14)
C7	0.0197 (15)	0.0200 (17)	0.0174 (15)	0.0032 (13)	0.0073 (13)	−0.0009 (13)
C8	0.0242 (17)	0.0265 (19)	0.0207 (17)	−0.0025 (14)	0.0061 (14)	−0.0018 (14)
C9	0.0270 (17)	0.0252 (19)	0.0230 (17)	0.0011 (15)	0.0092 (15)	0.0020 (14)
C10	0.0277 (18)	0.0211 (18)	0.0177 (16)	0.0030 (14)	0.0053 (14)	0.0024 (13)
C11	0.0211 (16)	0.0210 (17)	0.0173 (16)	0.0030 (13)	0.0047 (13)	0.0007 (13)
C12	0.0183 (15)	0.0171 (16)	0.0196 (15)	0.0055 (12)	0.0067 (13)	−0.0004 (13)
C13	0.0207 (16)	0.0243 (18)	0.0190 (16)	0.0008 (13)	0.0077 (13)	−0.0010 (14)
C14	0.0221 (16)	0.0233 (18)	0.0186 (16)	0.0010 (14)	0.0048 (13)	−0.0043 (13)
C15	0.0301 (19)	0.0168 (17)	0.0313 (19)	−0.0029 (14)	0.0048 (16)	0.0018 (15)
C16	0.031 (2)	0.028 (2)	0.036 (2)	−0.0106 (16)	0.0089 (17)	0.0005 (17)
C17	0.0294 (19)	0.028 (2)	0.037 (2)	−0.0022 (16)	0.0102 (17)	−0.0013 (17)
C18	0.028 (2)	0.046 (3)	0.062 (3)	−0.0073 (19)	0.016 (2)	−0.006 (2)

### Geometric parameters (Å, °)

**Table:**

Cd1—Br4	2.5168 (6)	C7—C8	1.394 (5)
Cd1—Br3	2.5665 (6)	C7—C12	1.479 (5)
Cd1—Br1	2.5720 (4)	C8—C9	1.369 (5)
Cd1—Br2	2.5901 (5)	C8—H8	0.9500
N1—C5	1.342 (4)	C9—H9	0.9500
N1—C9	1.348 (5)	C10—C11	1.370 (5)
N1—C4	1.489 (4)	C10—H10	0.9500
N2—C10	1.337 (4)	C11—C12	1.398 (4)
N2—C14	1.350 (4)	C11—H11	0.9500
N2—C15	1.492 (4)	C12—C13	1.384 (5)
C1—C2	1.523 (6)	C13—C14	1.371 (5)
C1—H1A	0.9800	C13—H13	0.9500
C1—H1B	0.9800	C14—H14	0.9500
C1—H1C	0.9800	C15—C16	1.531 (5)
C2—C3	1.505 (5)	C15—H15A	0.9900
C2—H2A	0.9900	C15—H15B	0.9900
C2—H2B	0.9900	C16—C17	1.497 (5)
C3—C4	1.507 (5)	C16—H16A	0.9900
C3—H3A	0.9900	C16—H16B	0.9900
C3—H3B	0.9900	C17—C18	1.521 (5)
C4—H4A	0.9900	C17—H17A	0.9900
C4—H4B	0.9900	C17—H17B	0.9900
C5—C6	1.366 (5)	C18—H18A	0.9800
C5—H5	0.9500	C18—H18B	0.9800
C6—C7	1.385 (5)	C18—H18C	0.9800
C6—H6	0.9500		
			
Br4—Cd1—Br3	106.22 (2)	C9—C8—C7	120.0 (3)
Br4—Cd1—Br1	106.574 (18)	C9—C8—H8	120.0
Br3—Cd1—Br1	119.113 (17)	C7—C8—H8	120.0
Br4—Cd1—Br2	105.991 (18)	N1—C9—C8	120.5 (3)
Br3—Cd1—Br2	106.390 (16)	N1—C9—H9	119.7
Br1—Cd1—Br2	111.718 (15)	C8—C9—H9	119.7
C5—N1—C9	120.8 (3)	N2—C10—C11	120.6 (3)
C5—N1—C4	119.5 (3)	N2—C10—H10	119.7
C9—N1—C4	119.7 (3)	C11—C10—H10	119.7
C10—N2—C14	120.8 (3)	C10—C11—C12	120.0 (3)
C10—N2—C15	119.2 (3)	C10—C11—H11	120.0
C14—N2—C15	119.9 (3)	C12—C11—H11	120.0
C2—C1—H1A	109.5	C13—C12—C11	117.8 (3)
C2—C1—H1B	109.5	C13—C12—C7	121.6 (3)
H1A—C1—H1B	109.5	C11—C12—C7	120.6 (3)
C2—C1—H1C	109.5	C14—C13—C12	120.2 (3)
H1A—C1—H1C	109.5	C14—C13—H13	119.9
H1B—C1—H1C	109.5	C12—C13—H13	119.9
C3—C2—C1	110.9 (4)	N2—C14—C13	120.5 (3)
C3—C2—H2A	109.5	N2—C14—H14	119.8
C1—C2—H2A	109.5	C13—C14—H14	119.8
C3—C2—H2B	109.5	N2—C15—C16	109.5 (3)
C1—C2—H2B	109.5	N2—C15—H15A	109.8
H2A—C2—H2B	108.0	C16—C15—H15A	109.8
C2—C3—C4	111.6 (3)	N2—C15—H15B	109.8
C2—C3—H3A	109.3	C16—C15—H15B	109.8
C4—C3—H3A	109.3	H15A—C15—H15B	108.2
C2—C3—H3B	109.3	C17—C16—C15	113.5 (3)
C4—C3—H3B	109.3	C17—C16—H16A	108.9
H3A—C3—H3B	108.0	C15—C16—H16A	108.9
N1—C4—C3	111.4 (3)	C17—C16—H16B	108.9
N1—C4—H4A	109.3	C15—C16—H16B	108.9
C3—C4—H4A	109.3	H16A—C16—H16B	107.7
N1—C4—H4B	109.3	C16—C17—C18	112.9 (3)
C3—C4—H4B	109.3	C16—C17—H17A	109.0
H4A—C4—H4B	108.0	C18—C17—H17A	109.0
N1—C5—C6	120.1 (3)	C16—C17—H17B	109.0
N1—C5—H5	119.9	C18—C17—H17B	109.0
C6—C5—H5	119.9	H17A—C17—H17B	107.8
C5—C6—C7	121.0 (3)	C17—C18—H18A	109.5
C5—C6—H6	119.5	C17—C18—H18B	109.5
C7—C6—H6	119.5	H18A—C18—H18B	109.5
C6—C7—C8	117.5 (3)	C17—C18—H18C	109.5
C6—C7—C12	121.4 (3)	H18A—C18—H18C	109.5
C8—C7—C12	121.1 (3)	H18B—C18—H18C	109.5
			
C1—C2—C3—C4	177.1 (4)	N2—C10—C11—C12	0.6 (5)
C5—N1—C4—C3	−89.0 (4)	C10—C11—C12—C13	−1.9 (5)
C9—N1—C4—C3	89.4 (4)	C10—C11—C12—C7	179.4 (3)
C2—C3—C4—N1	−178.1 (3)	C6—C7—C12—C13	−154.1 (3)
C9—N1—C5—C6	2.2 (5)	C8—C7—C12—C13	24.6 (4)
C4—N1—C5—C6	−179.4 (3)	C6—C7—C12—C11	24.5 (4)
N1—C5—C6—C7	0.4 (5)	C8—C7—C12—C11	−156.8 (3)
C5—C6—C7—C8	−1.8 (5)	C11—C12—C13—C14	1.8 (5)
C5—C6—C7—C12	176.9 (3)	C7—C12—C13—C14	−179.5 (3)
C6—C7—C8—C9	0.7 (5)	C10—N2—C14—C13	−0.9 (5)
C12—C7—C8—C9	−178.1 (3)	C15—N2—C14—C13	174.8 (3)
C5—N1—C9—C8	−3.4 (5)	C12—C13—C14—N2	−0.4 (5)
C4—N1—C9—C8	178.2 (3)	C10—N2—C15—C16	70.9 (4)
C7—C8—C9—N1	1.9 (5)	C14—N2—C15—C16	−104.9 (3)
C14—N2—C10—C11	0.8 (5)	N2—C15—C16—C17	61.5 (4)
C15—N2—C10—C11	−174.9 (3)	C15—C16—C17—C18	−176.9 (3)
